# Identification of new demands regarding prehospital care based on 35,188 missions in 2018

**DOI:** 10.1186/s12873-021-00456-w

**Published:** 2021-05-24

**Authors:** Séverine Vuilleumier, Assunta Fiorentino, Sandrine Dénéréaz, Thierry Spichiger

**Affiliations:** 1 La Source School of Nursing, University of Applied Sciences and Arts Western Switzerland (HES-SO), CH-1004 Lausanne, Switzerland; 2 Vocational Training College for Registered Paramedics and Emergency Care, ES ASUR, CH-1052 Le Mont-sur-Lausanne, Switzerland

**Keywords:** Prehospital, Emergency medical service, Non-urgent patients, Transport

## Abstract

**Background:**

Population ageing and increased prevalence of chronic diseases result in the emergence of new demands in prehospital care. The prehospital system is facing an increase of cases without acute threat to life (so-called “non-urgent”), which generates tension due to a higher number of admissions to emergency departments and a greater use of prehospital resources. Our aim is to understand this transition in prehospital activities and to delineate the primary missions performed by paramedics in 2018 with a focus on the population concerned, the severity of cases encountered and the typology of health issues.

**Method:**

The study is retrospective, and descriptive, using a statistical description of 35,188 primary missions realized in 2018 in the State of Vaud (Switzerland). The characteristics taken into consideration are the age and gender of patients, as well as the health issue, the severity of cases based on National Advisory Committee for Aeronautics score (NACA score), and the time and place of intervention.

**Results:**

The results describe the primary missions in the State of Vaud in 2018 and show that 87% of missions concern “non-urgent” situations (without acute threat to life). Over half of patients are 65 or older, the highest proportion of health issues, 49%, are medical and only 23% of missions are for traumas. Mission related to mental health issues reach 7% and those for intoxication 6%. Most missions take place between 7:00 am and 6:00 pm (67%), and around 12% of missions lead to the non-transport of the patient.

**Conclusion:**

The prehospital sector is confronted with a major transition in terms of patient care. An increase of non-urgent cases is observed, associated with the care of persons aged 65 or more. Our results question the adequacy between the needs in terms of prehospital care and the paramedic profession as it is currently defined, as well as the place of this profession within the health network. Reflecting upon the role of paramedics with respect to the socio-demographic evolution of populations appears necessary, to analyse the adequacy of the paramedics’ skills to respond to the current needs.

**Supplementary Information:**

The online version contains supplementary material available at 10.1186/s12873-021-00456-w.

## Background

One of the challenges of the Swiss health system is, like in all European countries, first to maintain accessibility to quality care for the entire population, and second to stop the growing increase in health costs [1]. The prehospital sector holds a predominant position in maintaining the population’s access to care. It provides continuous (24 h a day) and diversified care (maternity, diseases, traumas) to the entire population, and transports patients within health networks [1]. In 2015, paramedic services have performed 460,000 assistance and rescue missions throughout Switzerland [2].

Over the last decades, increased demand for emergency prehospital services has been observed in many developed countries [3]. This trend is also observed in the State of Vaud in the French speaking part of Switzerland [2]. For example, between 2010 and 2016, the total number of paramedic missions went from 40,075 to 48,431 [4]. This increase has been proposed to be linked to higher use of hospital emergency departments by senior patients [3, 5, 6], by non-life-threatening conditions [7–9] and by mental health issues [6]. In earlier studies, an increase in demand for emergency ambulance services has also been associated with factors such as facilitated access to ambulance services and health awareness [3]. Hospitals suffer from the repercussions of the number of ambulance transports, as admissions in emergency departments constantly increase and waiting times are extended. This increase has a direct impact on the quality of services of care and can have important consequences, like a higher number of deaths in emergency departments [10, 11]. This increase also leads to higher healthcare costs. In Switzerland, the budget dedicated to healthcare costs corresponds to 14.5% of the Gross Domestic Product, and is in second position worldwide, behind the United States [12]. New strategies must be found to restrict the financial impacts on health, especially in relation to population aging and to the increase in chronic diseases [13].

Over 20% of emergency department admissions occur via the use of prehospital services [14]. To control congestion in emergency departments due to cases that do not require urgent care, and therefore to reduce the admission rate, it is necessary to revise procedures and to rethink allocation of prehospital healthcare providers [15–17]. The role of paramedics, which was initially to respond to emergencies, is now faced with new health issues characterised by the fact that the prehospital patients’ health problems are often of low severity. Indeed,84.4% of primary missions in the State of Vaud were concerned by low severity cases in 2010 [2].

Paramedic missions are regulated at the State (canton) level [18]. They are based on algorithms allowing for an appropriate, efficient and coordinated response between the different healthcare providers involved, i.e. ambulances and mobile units with an emergency physician, in life-threatening situations such as cardiac arrests or respiratory failures [19]. These algorithms are inspired by international recommendations, which are regularly updated, and are essential for the collaboration of the different prehospital partners in order to determine which treatment and orientation are required for a patient [20]. They are however reaching their limits and are in fact not flexible enough for the assessment and treatment of non-urgent patients, who require complex and nuanced care.

The demographic and epidemiological evolution of the population leads to new health issues related for example to the population ageing and to the increased prevalence of chronic diseases, which have a direct impact on the prehospital system, and are responsible for an overload of emergency departments as well as a rise of health costs. Our goal is to better understand the challenges faced by the prehospital sector. More specifically, our study aims at identifying and quantifying 1) the proportions of primary missions with and without vital threats, and among them 2) the age distribution, 3) the health issues being addressed, 4) the localization and the hourly distribution of the missions, and finally 5) the proportion of non-transported patients. To do so, we analysed retrospectively all the primary missions performed in 2018 in the State of Vaud of Switzerland. This study provides an in-depth description of prehospital missions, of the typology of patients and of the health issues encountered.

## Methods

### Study design

This study is a retrospective, observational, and descriptive study realized on 35,188 primary missions performed by the prehospital services in 2018. Every mission is documented, and the collected data provides information on the patient (e.g. age, gender, health issue, severity of condition) and on the intervention (e.g. time or place). All this information has been entered by paramedics after each mission and stored in a database called “Attrib”. An anonymised dataset of the Attrib database has been made available by the Directorate-General for Health of the State of Vaud. All methods were performed in accordance with the relevant guidelines and regulations.

### Study setting

This study has been conducted in the State of Vaud in Switzerland which represents a population of around 800,000 inhabitants and where eight ambulance services are primarily active in rescue and assistance missions. The prehospital setting has four major partners: 1) emergency medical dispatch centres, 2) ambulance services, 3) mobile units (and helicopter) staffed with emergency physicians and 4) social or local partners affiliated to respond to emergencies. The ambulance services are directly dispatched by the 144 emergency medical dispatch centres, around the clock. The study focuses only on the primary missions that are regulated by the 144 emergency medical dispatch centres, and are emergency responses to an out-of-hospital location.

### Data

The data used for this study is for the period going from 1 January 2018 to 31 December 2018. The data were collected by the paramedic services and made available by the Directorate-General for Health of the State of Vaud. They were entered in the Attrib database during the same period. Only primary missions are analysed as secondary missions concern transfer between health institutions. The data was anonymised before the analysis. The data are: the age and gender of the patient, the time of intervention, the place of intervention, the NACA score, the principal health issue (problem code), and transport, see the detailed description below.

*The place of missions* has been divided into 7 categories (place of residence, public place, training or workplace, health institutions, mental health institutions, law enforcement institutions, and others), which gather the 26 types of place available in the Attrib database (Table S1 in Additional file 1). The nursing home and social institutions host around 6000 residents [21]. In the category “health institutions” the nursing home and social institutions as well as dialysis centre and medical practice are included.

*The NACA score* (see detailed description in Table S2 in Additional file 1) ranks the degree of severity of traumas and pathologies. It was created to score the condition severity of a patient 24 h after admission in hospital [22, 23]. The NACA score was then adapted for medical and surgical conditions in prehospital settings [24] and it is now widely used at the international level [25–27]. Studies validated NACA outcomes like mortality and morbidity or prediction to survival rate [23, 26, 27]. The NACA score is a scale with 8 values (0–7), the minimum score being 0 (unscathed patient) and the maximum 7 (deceased patient). A NACA score of 3 describes a health issue without an acute threat to life. It can be considered as the threshold indicating that a patient requires hospital investigation and treatment (as defined in its scale, Table S2), the trauma or pathology being non-life-threatening (e.g. fractured ankle). A NACA score of 4 means that without hospital treatment, the disease or lesions could evolve towards vital risk (for example a myocardial infarction). In this study, the cases with a NACA score between 4 and 7 are considered as “urgent” cases, and cases with NACA scores between 0 and 3 as “non-urgent”. Nevertheless, we are fully aware that the definition of “urgent” or “non-urgent” is debatable: a clinical state qualified as urgent means that it is necessary to act quickly, whereas a severe health issue does not necessarily require emergency treatment. The NACA score is assigned by the paramedics.

*The problem code* indicates the main health issue identified by the paramedics during the intervention, for example, trauma, cardiac arrest or intoxication. The health issue is described according to 33 “problem codes” (Table S3, in Additional file 1). Within the context of our study, the 33 problem codes have been gathered to form 6 categories: Trauma, Cardiac arrest, Medicine, Psychiatry, Toxicology and Other. Cardiac arrest is classified as a distinct category as it can be either traumatic or medical. The proposed categories are presented in (Table S3 in Additional file 1).

Finally, *transport* determines whether the patient was taken to hospital by the paramedics or not. When the patient is not transported, the cause of non-transport is registered among 12 defined categories which are the following: Ambulance Leader / Triage, Transport refused by the patient, Technical problems, Fire preventions, Police preventions, Patient not found, Patient left on site, Patient left to the police, Patient who will visit a doctor later on, Deceased patient, Mission cancelled on site, Mission cancelled on the way and Contacted doctor.

### Statistics

The variables analysed for all the missions were: the age and gender of the patient, the time of intervention, the place of intervention, the NACA score, the principal health issue (problem code), and transport. When information on a variable was missing for an intervention, it was excluded, but only when the analysis was on the concerned variable. The missing data are indicated in the analyses. The data have been analysed using sorting and filtering function and descriptive statistics including percentage, median, standard deviation (SD) and interquartile range (IQR) as well as distribution over time, and boxplots. Comparative statistics have used the Mann–Whitney U test. Boxplots and the statistical tests have been performed with the GraphPad Prism software version 7 [28]. Other descriptive statistics have been calculated with the Microsoft Office Excel 2019 software.

## Results

### Primary missions and NACA score

The number of missions in the State of Vaud in 2018 was 36,847, amongst which 1659 (4.5%) were excluded from the study. These latter cases correspond to cancelled missions or missions without contact with the patient. As for the remaining 35,188 (95,5%) primary missions, over 87% (*n* = 30,665) patients have a NACA score between 0 and 3 (non-urgent cases), amongst which around 47.3% (*n* = 16,629) a score of 3 (Table 1). NACA scores between 4 and 6 (urgent cases), which indicate a possible deterioration of the patient’s vital signs, represent 11.48% (*n* = 4040) of missions, and around 1.4% (*n* = 483) patients were declared as deceased (NACA score of 7) on the location of the mission.

**Table 1:** Number and percentage of NACA scores (*n* = 35,188) and distribution of patients per age group (*n* = 35,156, 32 undocumented age) in 2018 prehospital service of the State of Vaud

Number of Missions									
**NACA Score**	**0**	**1**	**2**	**3**	**4**	**5**	**6**	**7**	**Total**
	628 (1.78%)	2615 (7.43%)	10,793 (30.67%)	16,629 (47.26%)	2908 (8.26%)	937 (2.66%)	195 (0.55%)	483 (1.37%)	35,188
**Age group**		**0–16**		**17–64**		**65 +**		**Total**	
		2160 (6.14%)		15,193 (43.22%)		17,803 (50.64%)		35,156	

### Ages and NACA score

**Fig. 1:**
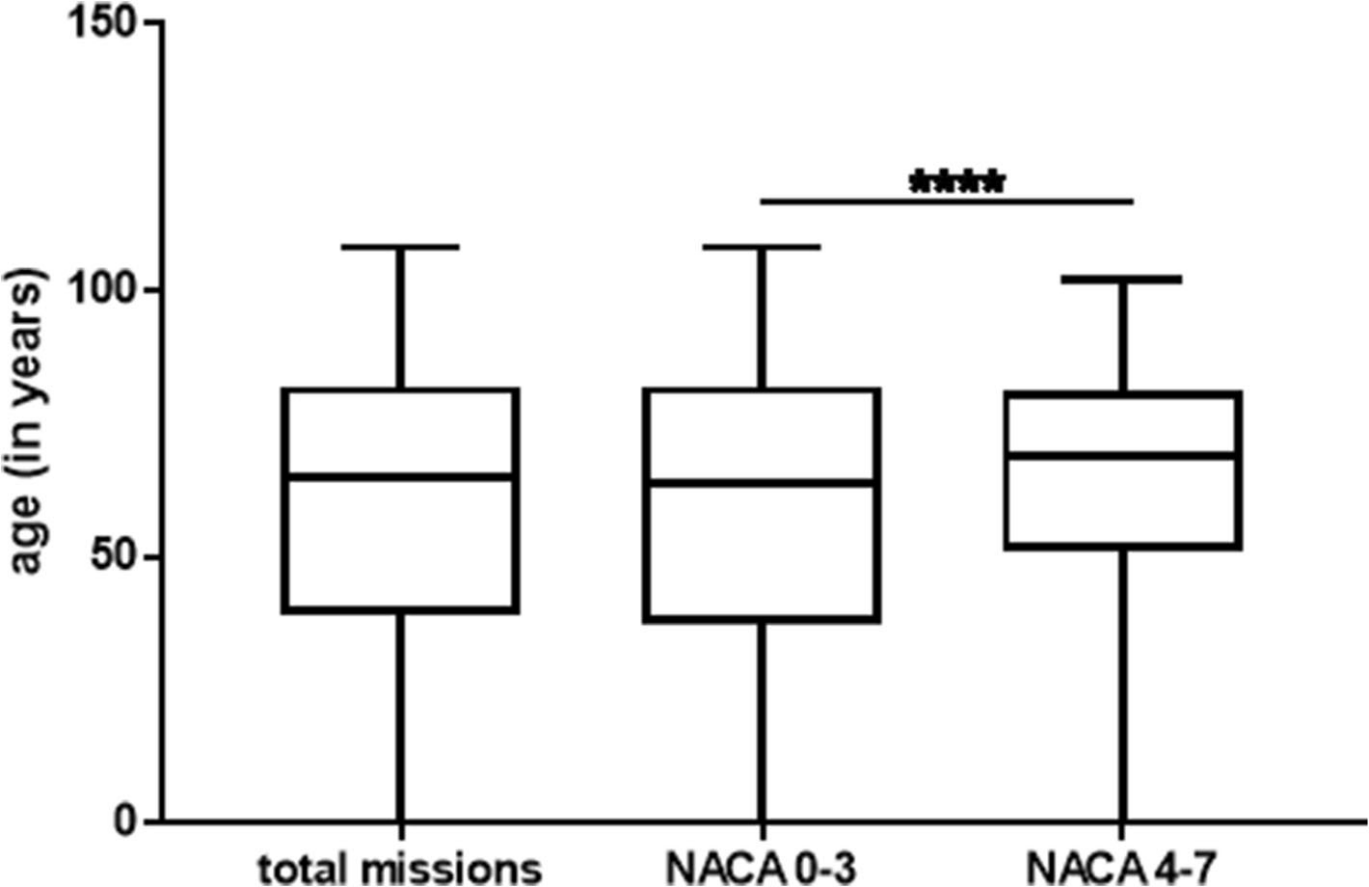
Age distribution as a function of the level of severity determined by the NACA score. Boxplot 1 represents all the missions in 2018 (*n* = 35,156, 32 data items missing). Boxplots 2 and 3 respectively represent the age distributions for the prehospital missions with NACA scores between 0 and 3 (non-urgent, *n* = 30,633) and NACA scores between 4 and 7 (vital emergency, *n* = 4523). The Mann–Whitney U test shows that the two medians are different with high significance. For NACA scores 0–3 and NACA scores 4–7, statistical values are respectively: median: 64–69, SD: 26.06–22.80, IQR: 45–30

The age distribution of the patients shows that 50.64% (*n* = 17,803) primary missions concern patients aged 65 or more, 43.22% (*n* = 15,193) are between 17 and 64 years old, and 6.14% (*n* = 2160) between 0 and 16 years old (Table 1). Persons aged 65 or more, therefore, represent most primary missions, the median being at 65 (Fig. 1, Boxplot 1). For NACA scores between 0 and 3, the median is at 64, whereas for NACA scores between 4 and 7, the median is at 69 (Fig. 1, Boxplot 2 and 3). The difference between these two groups is very significant (*p*-value < 0.0001, the Mann–Whitney U test). The proportion of men and women is similar with 51% (*n* = 17,850) women and 49% (n = 17,310) men (based on the documented data, *n* = 35,160, undocumented *n* = 28).

### The typology of health issues

**Fig. 2:**
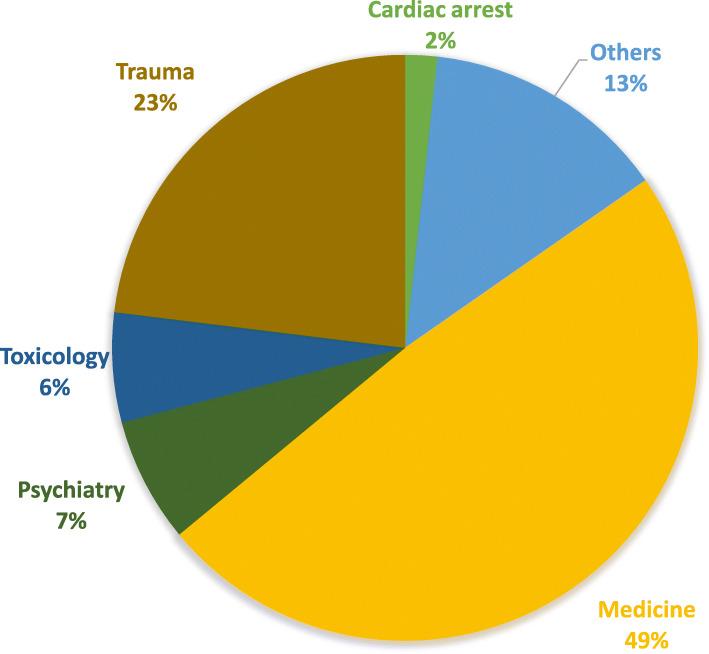
Typology of the health issues encountered in 2018 (*n* = 35,184–4 missing data)

Traumatic cases represent 23% (*n* = 8111) of the health issues encountered by the paramedics and around one out of two primary mission was for medical issues (49%, *n* = 17,131) (Fig. 2, Table S3), amongst which cases of respiratory distress or failure (around 7%, *n* = 2439), decrease in general health condition (around 8%, *n* = 2702), or brief loss of consciousness (around 6%, *n* = 1977). Mental health issues are found with similar proportions (7%, *n* = 2423), intoxications as well (6%, *n* = 2131). As for cardiac arrests, they represent 2% of all missions (*n* = 625). Finally, one should note that a large proportion of missions are in the “Other” category (around 13%, *n* = 4763) (Details on health issues encountered are presented in Table S3 in Additional file 1).

### Places and times of mission

**Fig. 3:**
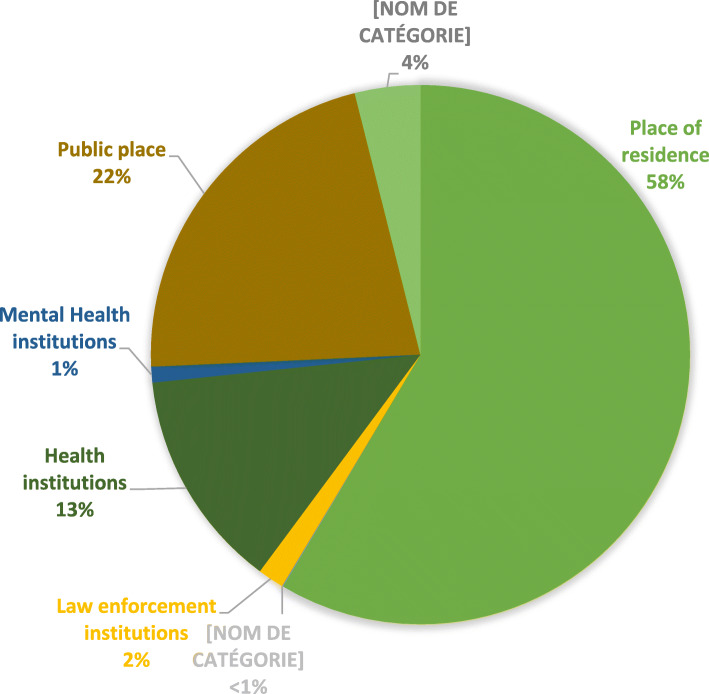
Places of intervention in 2018 (*n* = 35,188)

The patient’s place of residence is the main place where missions occur, with 58% (*n* = 20,598) of cases (Fig. 3). About 22% (*n* = 7667) of the missions were in public places and 13% (*n* = 4639) occur in a health institution. Training and workplaces represent 4% (*n* = 1380) of missions (details on places of mission are presented in Table S1 in Additional file 1).

**Fig. 4:**
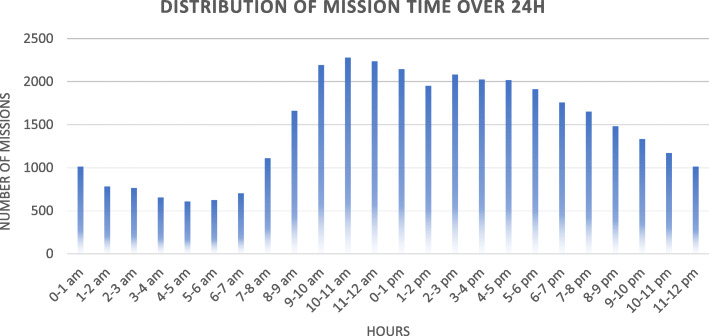
Distribution of missions of the year 2018 over the hour of the day (*n* = 35,188)

The distribution of missions over 24 h (Fig. 4) shows that a large proportion of missions occur between 7:00 am and 6:00 pm. Missions remain frequent until midnight (19% (*n* = 6653) occur between 6:00 pm and 0:00 am and 13% (*n* = 4458) between 0:00 and 6:00 am), then they considerably decrease (details on the number of mission time are presented in Table S4 in Additional file 1).

### Non-transported patients

A high number of non-transported patients (87.4%, *n* = 3612) have a NACA score between 0 and 3 (Table 2). One should note that 11.6% (*n* = 480) of the non-transported patients are patients who died on site (NACA 7). The age group mainly concerned by non-transport is that of persons between 17 and 64 (51.2%, *n* = 2104), and to a lesser extent, persons aged 65 or more (38.1%, *n* = 1566) (Table 2). Primary missions lead to the transport of 88.3% (*n* = 31,053) patients to a hospital facility, while 11.7% (*n* = 4135) do not. When there is no transport, 61.2% (*n* = 2529) of initial missions occur at the patient’s place of residence, 27.9% (*n* = 1152) in a public place or in the street, 5.6% at the workplace (233), and 3.2% (*n* = 131) in a health institution (Table 3). Amongst the non-transported patients, 46.3% (*n* = 1913) are left on site, 25.4% (*n* = 1050) will see a doctor at a later stage, and 5.1% (*n* = 210) have refused to be transported (Table 3).

**Table 2:** NACA score (*n* = 4135) and age (*n* = 4108, age not documented in *n* = 23 cases) of non-transported patients

Non-transported patients									
**NACA score**	**0**	**1**	**2**	**3**	**4**	**5**	**6**	**7**	**Total**
	568 (13.7%)	2174 (52.6%)	796 (19.3%)	74 (1.8%)	22 (0.5%)	19 (0.5%)	2 (< 0.01%)	480 (11.6%)	4135
**Age group**		**0–16**		**17–64**		**65 +**		**Total**	
		438 (10.7%)		2104 (51.2%)		1566 (38.1%)		4108

**Table 3:** Events (*n* = 4135) and places of missions (*n* = 4131, 4 cases not documented) leading to a non-transport

Non-transport			
Events leading to a non-transport	Number (%)	Places of missions	Number (%)
Ambulance Leader / Triage	8 (0.2%)	Training and workplace	233 (5.6%)
Transports refused by the patient	210 (5.1%)		
Technical problems	5 (0.1%)	Public place	1152 (27.9%)
Firemen preventions	3 (0.1%)	Mental health institution	5 (0.1%)
Police preventions	1 (0.0%)	Health institution	131 (3.2%)
Patients not found	2 (0.0%)		
Patients left on site	1913 (46.3%)	Law enforcement institution	37 (0.9%)
Patients left to the police	229 (5.5%)	Place of residence	2529 (61.2%)
Patients who will visit a doctor later on	1050 (25.4%)		
Deceased patients	479 (11.6%)	Medical centre	42 (1.0%)
Missions cancelled on site	9 (0.2%)		
Mission cancelled on the way	1 (0.0%)	Others	2 (< 1%)
Contacted doctors	225 (5.4%)		
Total	4135	Total	4131

## Discussion

Our results show that 87% of the missions in 2018 can be classified as “non-urgent” and that over half of the patients are 65 or older. Nearly 12% of ambulance calls lead to the non-transport of the patient, and only 23% of missions deal with trauma. While responding to vital emergencies is a priority in paramedics’ scope of practice, our results suggest that the actual needs of the population requesting an ambulance are much wider, and mainly concern non-vital prehospital care for an increasingly aging population and for patients presenting a low severity score. The adequacy between the population needs in terms of prehospital care and the profession of paramedic as it is currently defined in Switzerland is discussed below, as well as the integration of ambulance services within the healthcare network.

### A challenge: the increase in the number of missions for “non-urgent” cases

#### More prehospital missions without acute threat to life

. The number of primary missions in the State of Vaud in 2018 increased by over 22%, compared to 2010 [2]. This increase reflects a general rise in the number of (primary and secondary) paramedic missions in the State of Vaud: they have increased by 50% since 2005, going from 33,920 to 48,807. In 87.2% of cases, missions concern patients with NACA scores going from 0 to 3, i.e. “non-urgent” cases. These numbers confirm the trend already observed in 2003, then in 2010, as there were respectively 82.8 and 84.4% “non-urgent” missions during these years [2]. High proportions of non-urgent cases have also been recorded in other countries, for example in Norway [29] and the United Kingdom [30].

#### Senior patients

Our results show that senior persons highly request prehospital services, which confirms the results already observed between 2001 and 2010 [2]. This trend is also found internationally, as shown by a study conducted in the United-States in North Carolina, which indicates that transport consumption by patients aged 65 or more represents 44% (*n* = 1,719,998) of all ambulance transports [5]. This population displays clinical specificities, such as risk factors that are more important and comorbidities requiring specific skills and knowledge (clinical assessment of senior persons or geriatric syndromes) [15, 31]. Alternatives to ambulance transport should be sought [2] especially in regards to projections that estimate that there will be 2.7 million people aged 65 or older by 2050 in Switzerland, whereas 1.6 million are counted at the end of 2019 [32].

#### Place and time of intervention

Our results show that most primary missions occur at the patients’ place of residence, as was already the case in 2010 [2]. They highlight an important number of primary intervention requests, reaching 2628, coming from medical-social institutions. In 2013, this number was 1898. It was already considered as important and explanations for it was the existence of patient previous medical conditions and the fact that there was no primary care physician on site [17]. As in our study, most missions concerned cases having NACA scores between 0 and 3.

The distribution of missions over 24 h in 2018 (Fig. 4) shows that most demands for an ambulance take place between 7:00 am until 6:00 pm, then gradually decrease until midnight with few missions between 2:00 and 7:00 am. An Australian study shows a similar distribution of missions over 24 h, with a downward curve from 7:00 pm [30]. With 63% of missions occurring between 7:00 am and 6:00 pm, our results are close to those documented by Pittet et al. in 2014, who found that 62% of emergency medical dispatch centre calls were between 7:00 am and 7:00 pm [2]. However, our results show that 2 missions out of 10 occur between 6:00 pm and midnight. This reality, which is also observed in other studies, raises questions linked to the allocation of prehospital resources that are adapted to needs over 24 h [30]. A recent recommendation of the National Health Services (UK) advises staggering paramedics’ working hours when teams rotate, more specifically during the first hours of the evening [33]. Complementary investigations with healthcare providers would be necessary to better understand the adequacy of their allocation through time.

### Patients left at their place of residence: a delicate balance

#### One person out of ten is not transported

Our study shows that 11.7% of patients who requested paramedic services were not transported to a care institution. In around 72% of cases, the patient was left on site or went to visit a doctor later. The large majority (46.3%) of non-transported patients are “left on site”. In the State of Vaud, this decision is taken by the paramedics in agreement with the patient’s will, following an assessment of the patient’s health condition. Patients are informed about possible deterioration signs and symptoms due to their condition and taught to call the emergency medical dispatch centre if they were to develop. Unfortunately, little is known about those patient’s outcomes, as discussed later.

These numbers may seem high, but they are in fact much lower than those documented in other countries. For example, a study conducted in England mentions a percentage of 29% for non-transports, following the patient’s assessment by the paramedics [34]. This number goes up to 37.7% in Finland [35] and 31.2% in the Netherlands [36]. One should note that according to Evans et al., out of 6559 senior patients who were not transported to hospital, only 19.7% were transported by the paramedics within the 30 days following the initial call [5]. In another study, 25.3% of non-transported patients called the emergency medical dispatch centres within hours or days after the initial call, amongst which 46.8% were finally taken to hospital [35]. To better understand the State of Vaud’s numbers, it would be necessary to examine whether they reflect i) a particularly high performance of the regulation before the intervention (144 calls), ii) the paramedics’ systematic overestimation of the severity of the health issue leading to transport, iii) a no risk approach by the paramedics, linked to uncertainty during triage, which boosts transports to emergency departments, or iv) a lack of coordination between the different healthcare providers concerning the next steps to follow for the care of patients who do not require transport to emergency departments but could benefit from a health network.

The hypothesis of a particularly high performance of the regulation by the 144 emergency medical dispatch centre compared to other European dispatch centres should probably be ruled out. Indeed, studies converge on showing an overestimation of the degree of severity, a trend which is in fact desirable in a health system founded on patient safety. In the State of Vaud, it has been shown that the dispatcher (registered paramedics and nurses with at least 5 years of field experience) overestimates the severity of the issue compared to the NACA score finally assigned by the paramedic [25]. This overestimation concerns 78% of calls for NACA scores < 4. Similar results were obtained in two French-speaking dispatch centres in Switzerland. Overestimations is also observed in other countries [37] however, these results are difficult to generalise, as the severity scales used might differ.

The question of paramedics’ overestimation of health issues, which leads to unnecessary transport, is expected to be related to the ability or difficulty in assessing the patients’ needs, the uncertainty on the risks run by a patient who is not transported and the will to avoid risk [38]. In the countries where non-transport is close to 30%, many alternatives are proposed to respond to emergencies and advanced practice exists to limit unnecessary transports [30, 39]. Differences in patients’ initial health condition, in paramedics’ experience, in local procedures and in education and training might then have an impact on the paramedics’ performance to accurately triage a patient. In Switzerland, paramedics graduate after 3 years in a vocational training college, whereas in other countries like England their education is at a bachelor’s degree level. Additional investigations on the possible gap between paramedic education and what is experienced during missions would be necessary to identify potential adaptation needs.

A lack of coordination between the different healthcare providers (with a palliative care network, for example) could lead to unnecessary transport to a health institution [40–42]. Our results show that only around 1% of non-transported patients had a NACA score between 4 and 6. This low proportion may reflect a lack of options for paramedics to organise alternative care. For example, a Canadian study showed the benefit of implementing a palliative care programme for paramedics. This programme avoids hospitalisation in an emergency department, amongst other things, and helps to increase families’ satisfaction [40].

In the State of Vaud, paramedics cannot leave a patient at his or her place of residence after they have performed a medical treatment, contrary to the “*treat and release*” practice applied in other countries [43]. This could partially explain the different percentages for non-transported patients compared to other countries. To better understand the issue linked to the non-transport of patients in the State of Vaud, it would be necessary to have a detailed description of what happens to them afterwards, such as the rates of under-triage and over-triage resulting from the patient’s assessment by the paramedics. Further research would also be necessary to better estimate the consequences of a higher level of paramedics’ autonomy on the entire prehospital setting.

#### Few senior persons in non-transports

Amongst the non-transported patients, the most represented age group is aged between 17 and 64 (51.2%); persons aged 65 or more represent lower proportions (38.1%). Similar values have been found in Finland, where non-transport concerns patients over 69 in 43.4% of cases, and patients aged 80 or more in only 17% of cases [35]. This means that senior persons are proportionally more frequently transported to an emergency department. One of the most probable explanation is that it is difficult to exclude a severe health issue for this population, because of a high prevalence of comorbidities. This has been shown in other studies where the presence of comorbidities or depression explained the transport of the elderly [5, 44].

### Skills inadequacy for the current needs

#### Looking for alternatives to respond to the population’s needs

The drastic increase in “non-urgent” missions represents a major challenge for emergency departments. Indeed, the care of non-urgent patients can lead to a saturation of hospital emergency services, and also restricts the availability of emergency departments’ resources for urgent cases [25].

Vital emergencies are a care priority in paramedics’ specifications. To ensure a high level of performance in the care of vital emergencies, paramedics are trained to respond to different types of emergencies, for example those defined by the European Resuscitation Council [45, 46]: (a) cardiac arrest, (b) acute coronary syndrome, (c) acute stroke, (d) acute respiratory distress and (e) polytrauma or severe cervical head trauma. To this end, Swiss paramedics use specific algorithms as a support, which were developed for well-defined health issues, like for example cardiac arrests [47] or convulsions [48]. These algorithms are very effective and guarantee that the care provided for vital emergencies is coordinated between the different care providers [49]. As the “Others” category of the Attrib database represents 13% of health issues, it appears difficult to categorise precisely all the patients assessed by the paramedics. This shows the difficulty of assigning a rigorous problem code in complex situations. The results could also not represent all the pathologies reported by the practitioners in a comprehensive and adequate way.

Our results show that vital emergencies only concern 13% of missions: the remainder, i.e. 87%, concerns non-urgent missions (NACA scores between 0 and 3). The latter are, amongst others, for rather senior persons with complex pathologies (multiple diseases or chronic diseases) after a trauma, or a non-specific problem such as decreased general health condition (7.7%), or mental health cases (6.9%). These issues cannot be treated using algorithms, as they require a complex clinical assessment and diagnosis. The inadequacy of algorithms for non-urgent but complex cases was reported in several studies, for example in Gray et al. 2007, who show that in the United-Kingdom, algorithms are inappropriate in 39.4% of cases.

The discrepancy between training, the skills and the actual scope of practice in light of the actual missions (needs of the population) has a direct impact on the security of patients [35, 50]. To overcome this problem, some countries have developed triage procedures which are deployed by the paramedics on site [38, 51–54]. In Switzerland too, studies highlight the need to revise procedures and rethink the training for prehospital healthcare providers [2, 15, 55]. They show in particular the importance of developing skills linked to geriatric clinical assessment, as well as to decision-making and ethical approaches [55] which are expected to reduce hospital admissions and increase patient satisfaction [56].

### Toward a more diverse prehospital setting

Our study suggests that the actual challenge for paramedics is to take care of low-severity patients with complex needs. The solution might be to rethink the training for prehospital healthcare providers. Also, the inclusion of more diversified services in the health network that is centred on the needs and expectations of the patient and his or her family is an important area of research to explore. In the State of Vaud, some innovative ways of expanding health services are currently investigated. Among them, the exploration of the added value of having a health community nurse directly at home to evaluate the patient’s needs and to coordinate the health network around the patient, or the incorporation of social emergencies mobile teams are currently evaluated. Other innovative ways of expanding health services to accommodate the evolution of health needs, might emerge in regard to the digital transition of our societies.

### Limitations

Although our results are linked to the prehospital setting and how paramedics are trained in the State of Vaud, they represent well situations found in other States of Switzerland as well as in other countries. Main differences might occur when prehospital setting strongly differ, or when paramedic education level is higher. Regarding the data, the main limitation of the study might be related to the assessment of the patients’ health issue (problem code) and severity score (NACA score). Both are subjective processes that are expected to differ from one individual to another [57]. Moreover, problem codes might not describe well some health issues, and this is signalled by the high rate (13,5%) of codification of problem code as “others”. Also, errors might occur during the data collection due to inattention or difficulty to fulfil the required field in the database.

## Conclusion

The prehospital system must be adapted to ensure an optimal use of resources to respond to the needs, and to provide an adequate orientation of the patient within the health system. While paramedics need to maintain their skills to treat vital threats at the best level of care and in accordance with international standards, the actual challenge for them might be to take care of low-severity patients with complex needs, safely leaving them at home when appropriate or referring them to the right healthcare network.

## Supplementary Information

**Additional file 1: Table S1.** Place of mission and their aggregations into categories. **Table S2.** NACA scores and their signification used by Swiss emergency medical systems as originally described by the National Advisory Committee on Aeronautics (see [22]). It classifies severity level from NACA 0 (no injury) to NACA 7 (death). **Table S3.** Health issues encountered and their aggregations into categories. **Table S4.** Distribution of mission time over 24 h.

## Data Availability

The data that support the findings of this study are available from the Directorate-General for Health of the State of Vaud (Direction Générale de la Santé) but restrictions apply to the availability of these data, which were used under license for the current study, and so are not publicly available. Data as presented here in a yearly aggregated form are however available from the corresponding authors upon reasonable request and with permission of the Directorate-General for Health of the State of Vaud. All raw data belong to a database called “Attrib”, which provides an inventory of all the primary and secondary missions of the State of Vaud. Request about them should be send to the Directorate-General for Health of the State of Vaud .

## Abbreviation

NACA score: National Advisory Committee for Aeronautics score
