# Supplementary material for: Identification of new demands regarding prehospital care based on 35,188 missions in 2018
Source: BMC Emerg Med. 2021 May 24;21:63. doi: 10.1186/s12873-021-00456-w (PMC8142491; doi:10.1186/s12873-021-00456-w)
Supplement: Supplementary file 1 — Additional file 1: Table S1. Place of mission and their aggregations into categories. Table S2. NACA scores and their signification used by Swiss emergency medical systems as originally described by the National Advisory Committee on Aeronautics (see [22]). It classifies severity level from NACA 0 (no injury) to NACA 7 (death). Table S3. Health issues encountered and their aggregations into categories. Table S4. Distribution of mission time over 24 h. [file 12873_2021_456_MOESM1_ESM.docx]

**Additional file 1**

**Title: Identification of new demands regarding prehospital care based on 35,188 missions in 2018**

**List of authors:**

**Séverine Vuilleumier^1*^,**

**Assunta Fiorentino^1^,**

**Sandrine Dénéréaz^2^,**

**Thierry Spichiger^2^,**

^1^ La Source School of Nursing, University of Applied Sciences and Art Western Switzerland (HES-SO), CH-1004 Lausanne [s.vuilleumier@ecolelasource.ch](mailto:s.vuilleumier@ecolelasource.ch); a.fiorentino@ecolelasource.ch

^2^ES ASUR, Vocational Training College for Registered Paramedics and Emergency Care, CH-1052 Le Mont-sur-Lausanne [s.denereaz@es-asur.ch](mailto:s.denereaz@es-asur.ch), t.spichiger@es-asur.ch

*corresponding author: s.vuilleumier@ecolelasource.ch

**Table S1: Place of mission and their aggregations into categories**

| Place of mission | Number of cases | % | Category |
| --- | --- | --- | --- |
| Place of residence | 20598 | 58.5 | Place of residence |
| Other | 12 | 0.0 | Other |
| Airport-Heliport (drop zone) | 10 | 0.0 | Other |
| Prison facility | 305 | 0.9 | Law enforcement institution |
| Police station | 215 | 0.6 | Law enforcement institution |
| Parade ground | 24 | 0.1 | Law enforcement institution |
| Zone, regional hospital | 4 | 0.0 | Law enforcement institution |
| Clinic | 2 | 0.0 | Health institutions |
| University hospital | 1 | 0.0 | Health institutions |
| Medical permanence | 688 | 2.0 | Health institutions |
| Medico-social institution | 2628 | 7.5 | Health institutions |
| Treatment and rehabilitation centre | 7 | 0.0 | Health institutions |
| Institution and foundation | 597 | 1.7 | Health institutions |
| Medical practice | 680 | 1.9 | Health institutions |
| Outpatient surgery centre | 10 | 0.0 | Health institutions |
| Dialysis centre | 26 | 0.1 | Health institutions |
| Psychiatric hospital | 8 | 0.0 | Mental health institution |
| Psychiatric permanence | 326 | 0.9 | Mental health institution |
| Public road | 4279 | 12.2 | Public place |
| Motorway | 252 | 0.7 | Public place |
| Public place | 2400 | 6.8 | Public place |
| Sports and leisure | 736 | 2.1 | Public place |
| Training and workplace | 1380 | 3.9 | Training and workplace |
| Total | 35188 | 100.0 |  |

**Table S2: NACA scores and their signification used by Swiss emergency medical systems as originally described by the National Advisory Committee on Aeronautics (see 22). It classifies severity level from NACA 0 (no injury) to NACA 7 (death)**

|  |  |
| --- | --- |
| NACA 0 | No injury or disease |
| NACA 1 | Injuries/diseases without any need for acute physicians’ care |
| NACA 2 | Injuries/diseases requiring examination and therapy by a physician, but hospital admission is not indicated. |
| NACA 3 | Injuries/diseases without acute threat to life but requiring hospital admission. |
| NACA 4 | Injuries/diseases which can possibly lead to deterioration of vital signs. |
| NACA 5 | Injuries/diseases with acute threat to life. |
| NACA 6 | Injuries/diseases transported after successful resuscitation. |
| NACA 7 | Lethal injuries or diseases (with or without resuscitation attempts) |

**Table S3: Health issues encountered and their aggregations into categories**

| Problem code | Number of cases | % | Category |
| --- | --- | --- | --- |
| Cardiac arrest | 625 | 1.78 | Cardiac arrest (ACR) |
| Non-traumatic coma | 167 | 0.47 | Medicine |
| Alertness disorders | 766 | 2.18 | Medicine |
| Brief loss of consciousness, non cardiac attack (vasovagal response, hypotension) | 1977 | 5.62 | Medicine |
| Convulsive seizure | 917 | 2.61 | Medicine |
| Respiratory distress or failure | 2439 | 6.93 | Medicine |
| Asthma attack | 36 | 0.10 | Medicine |
| Heart attack, non-traumatic chest pain (conscious victim) | 1628 | 4.63 | Medicine |
| State of shock (cardiogenic, hypovolemic, septic, anaphylactic) | 264 | 0.75 | Medicine |
| Haemorrhage without trauma (digestive, otolaryngological, gynaecological) | 582 | 1.65 | Medicine |
| Rhythm and/or conduction disorders (bradycardia, av block, etc.) | 460 | 1.31 | Medicine |
| Hypertensive emergency | 306 | 0.87 | Medicine |
| Neurological deficiency without coma and non-traumatic | 911 | 2.59 | Medicine |
| Headaches | 336 | 0.95 | Medicine |
| Psychiatry (agitation, anxiety, etc.) | 2423 | 6.89 | Psychiatry |
| Intoxication without coma (alcohol, legal and illegal drugs, co, smoke) | 2131 | 6.06 | Toxicology |
| Allergy (without anaphylactic shock) | 248 | 0.70 | Medicine |
| Polytrauma | 130 | 0.37 | Trauma |
| Limb trauma (including dislocations) | 4347 | 12.36 | Trauma |
| Brain trauma | 1616 | 4.59 | Trauma |
| Maxillo-facial trauma | 647 | 1.84 | Trauma |
| Spinal column trauma | 648 | 1.84 | Trauma |
| Thoracic trauma | 424 | 1.21 | Trauma |
| Abdominal trauma | 88 | 0.25 | Trauma |
| Pelvic trauma (perineum) | 108 | 0.31 | Trauma |
| Non-traumatic abdominal pains | 1987 | 5.65 | Medicine |
| Non-traumatic lower back pains | 658 | 1.87 | Medicine |
| Care impossible at home | 612 | 1.74 | Medicine |
| Decrease in general health condition | 2702 | 7.68 | Medicine |
| Pregnancy, delivery, birth | 135 | 0.38 | Medicine |
| Burn | 61 | 0.17 | Trauma |
| Drowning without cardiac arrest | 6 | 0.02 | Trauma |
| Electric shock without cardiac arrest | 8 | 0.02 | Trauma |
| Hypothermia without cardiac arrest | 28 | 0.08 | Trauma |
| Other | 4763 | 13.54 | Others |
| Total | 35184 | 100.00 |  |

**Table S4: Distribution of mission time over 24 hours.**

| **Time** | **Number of Missions** |
| --- | --- |
| 0-1 am | 1015 |
| 1-2 am | 784 |
| 2-3 am | 767 |
| 3-4 am | 656 |
| 4-5 am | 610 |
| 5-6 am | 626 |
| 6-7 am | 705 |
| 7-8 am | 1113 |
| 8-9 am | 1661 |
| 9-10 am | 2193 |
| 10-11 am | 2279 |
| 11-12 am | 2236 |
| 12 am-1 pm | 2145 |
| 1-2 pm | 1951 |
| 2-3 pm | 2082 |
| 3-4 pm | 2025 |
| 4-5 pm | 2017 |
| 5-6 pm | 1912 |
| 6-7 pm | 1758 |
| 7-8 pm | 1651 |
| 8-9 pm | 1481 |
| 9-10 pm | 1333 |
| 10-11 pm | 1173 |
| 11-12 pm | 1015 |
| Total | 35188 |
